# A model to follow? The EU and global eco-social policy

**DOI:** 10.1177/14680181241246935

**Published:** 2024-04-25

**Authors:** Francesco Laruffa, Frank Nullmeier

**Affiliations:** University of Geneva, Switzerland; University of Bremen, Germany

**Keywords:** Economization, eco-social policy, European Social Model, global justice, global social policy

## Abstract

In this article, we study the political project promoted by the European Commission (EC) for tackling simultaneously socioeconomic and environmental issues. Based on a detailed analysis of the most relevant EC policy documents (adopted between 2000 and 2020) that explicitly articulate ecological and socioeconomic questions, we offer two contributions to the literature on eco-social policy. First, we identify the nature of what we call the ‘European Eco-Social Model’. This political project subordinates social-ecological goals to the economic rationality of growth, competitiveness and profits and de-politicizes the efforts to promote more sustainable societies and economies. Second, we show how the Commission is repositioning itself as a global leader in the transformation to sustainability, attempting to extend its particular eco-social model to the whole world. Overall, we argue that this ‘model’ is based on self-contradictory assumptions and cannot demonstrate how it should be able to solve problems of social inequality and climate change on a global level.

## Introduction

The concept of ‘European Social Model’ has attracted a lot of scholarly attention (e.g. Alber, 2010; Ferrera et al., 2001; Hermann and Hofbauer, 2007; Offe, 2003; Scharpf, 2002). In this article, we follow Jepsen and Serrano Pascual (2005: 238–239), arguing that the European Social Model involves a *political project* centred on defining specific policies that attempt to solve problems that are constructed as common to all European countries: It is a ‘social and political construct’ developed in the political discussion on ‘how to deal with current socio-economic challenges’. Crucially, at the European level, the expansion of the social often occurs through its ‘economization’: Social goals are promoted but reframed as economic goals (e.g. Laruffa, 2021). Thus, the European social dimension usually entails a ‘productivist, economised vision of social policy’ (Carmel, 2005: 51).

In this article, we study how the European Commission develops a comprehensive ‘*European Eco-Social Model*’, exploring the way in which the Commission discursively constructs a political project for dealing simultaneously with socioeconomic and ecological challenges. There is a growing interest in how social and ecological issues are framed together in the literature on eco-social policies and sustainable welfare (e.g. Bohnenberger, 2020; Gough, 2022; Koch and Fritz, 2014; Mandelli, 2022; Zimmermann and Graziano, 2020). Here, we want to contribute to the literature on eco-social policies in two ways. First, we investigate the nature of what we call the ‘European Eco-Social Model’, through an analysis of the discourse promoted by the EC on the policies that should address the socioeconomic and environmental challenges at the same time. Second, we show how the Commission is repositioning itself as a global leader in the sustainability transformation, attempting to extend its eco-social model to the whole world, thereby profoundly shaping the emerging paradigm of ‘global eco-social policy’ (Kaasch and Schulze Waltrup, 2021).

The remainder of the article is organized as follows. The next section briefly describes the data and methodology used. The central part of the paper presents the empirical analysis. Finally, in the conclusion, we discuss the results obtained, arguing that the ‘European Model’ is based on self-contradictory assumptions and cannot demonstrate how it should be able to solve problems of social inequality and climate change on a global level.

## Empirical basis and methodological approach

In order to understand the nature of the European Eco-Social Model, we focused our empirical research on the analysis of the policy discourse of the EC. While not necessarily the most powerful, the Commission is probably the most important actor when it comes to speak in the name of Europe as a whole (arguably, the Council is the most powerful body in the EU architecture, but this is also the most intergovernmental and thus the least supranational or genuinely ‘European’ institution). Following Mandelli’s (2022) definition of eco-social policies as ‘public policies *explicitly* pursuing both environmental and social policy goals in an *integrated* way’ (p. 340, emphasis in original), we included in the analysis the most important documents (starting with the ‘growth strategies’) adopted by the EC, which explicitly articulate the social and environmental dimensions in an integrated way. We tried to be exhaustive, that is, to cover *all* most relevant documents, working for example with inter-documents citations (i.e. including a text that, being cited in a document already under analysis, seems important). We stopped to add documents to our dataset when we noticed that no new important information could be gained by further incorporations, i.e. that enough information was collected to draw conclusions and that further inclusions would not have produced value-added insights (data saturation). In this context, we decided to confine the analysis to a period of 20 years (between 2000 and 2020) merely for symbolic/stylistic reasons (i.e. other documents with similar contents can be found outside this period). Also, we were not so much interested in a discussion of the evolution of the Commission’s discourse over this period. Thus, we did not focus on discursive changes. Our aim was to identify the main features of the European Eco-Social Model and to show how the Commission attempts to reposition it as a model for the whole world. While it is certainly true that with the European Green Deal approved in 2019 the Commission ‘prioritises environmental challenges like never before’ (Mandelli et al., 2022: 87; Schunz, 2022), the fundamental logics at play and the core definitional features of the European Eco-Social Model – as we will see – remain rather constant. Similarly, it is true that the Commission is not a homogeneous or isolated actor: each time, the documents have been written by different people under the influence of and through interaction with other institutional players. Once again, we were not so interested in investigating how the documents have been elaborated, which the relevant actors (e.g. who drafted them), and through which processes (e.g. negotiations with other actors) they have been adopted. Rather, focusing on a careful reading of the texts as they are, our intention was to elaborate on the *remarkable continuity of the Commission’s position* and to work out the structure of an always recurring solution for very different problem situations: the solution of economization. These choices imply some limitations. For example, on the basis of our empirical work, we are unable to assess whether economization represents a discursive strategy of the Commission for building consensus and a political coalition among actors with different worldviews and interests or if it entails a system of beliefs internalized by the Commission’s civil servants. In the same vein, we cannot uncover the mechanisms that led economization to prevail over alternative discourses. All these open questions could be the object of further research.

The relevance of investigating ideas and discourse for studying policies is by now well acknowledged in the literature. To perform the analysis, we draw methodologically from the fields of ‘Interpretive Policy Studies’ and ‘Critical Policy Studies’ (Bevir and Rhodes, 2016; Fairclough, 2013; Fischer, 2016, 2021; Fischer et al., 2017; Heinelt and Münch, 2018; Howarth, 2010; Jessop, 2010; Wagenaar, 2011; Nullmeier, 2018). These intertwined fields of research focus on ideas and discourse for studying policy paradigms and emphasize that deliberately abstaining from critique has the consequence of a lack of self-reflection on scientific work. If we construct variables that draw on concepts that have not been scrutinized, criticized and questioned with regard to their precise meaning and their relation to other concepts, we run the risk of adopting or creating terminology whose situatedness in overall discourse we are not aware of or take for granted. Both are instances of a naïve and insufficiently reflected mode of operation. In many respects, critique (conceptual scrutiny and reflective conceptual construction) is therefore a prerequisite of descriptive and explanatory research. But ‘critique’ also refers to the fact that a normative commitment to progressive changes aimed at overcoming domination and injustice informs the research process. From this perspective, we study how the Commission problematizes the current situations and develops policy solutions, interrogating the extent to which this problematization and these solutions obscure other policy approaches that could lead to different, perhaps more emancipatory futures.

## From the European Social Model to the European Eco-Social Model

In the early 2000s, the European Commission (EC, 2001) proposed a new approach to policymaking aimed at integrating socioeconomic and ecological concerns in a ‘comprehensive’ and ‘cross-sectoral’ way (p. 9). Uncoordinated policy actions were criticized because improvements in one policy area hindered progress in another. Moreover, ‘the absence of a coherent long-term perspective’ encouraged a focus on ‘short-term costs’ rather than on longer-term ‘win-win’ solutions (EC, 2001: 5). Building on this observation, the Commission elaborated ‘cross-cutting proposals and recommendations’, with sustainable development becoming ‘the central objective of all sectors and policies’ (EC, 2001: 5–6). Emphasizing the ‘environmental dimension’ of the European project implied a ‘positive long-term vision’ whereby ‘economic growth, social cohesion and environmental protection must go hand in hand’ (EC, 2001: 2).

This vision was confirmed and reinforced with the adoption of the Europe 2020 Strategy in 2010 and, most recently, with the adoption of the Green Deal in 2019. In the Europe 2020 Strategy, the ‘vision of Europe’s social market economy for the 21st century’ is articulated in terms of a ‘smart, sustainable and inclusive economy delivering high levels of employment, productivity and social cohesion’ (EC, 2010: 3). *Smart growth* implies ‘developing an economy based on knowledge and innovation’; *sustainable growth* entails ‘promoting a more resource efficient, greener and more competitive economy’; and *inclusive growth* requires ‘fostering a high-employment economy delivering economic, social and territorial cohesion’ (EC, 2010: 8). The Commission considers these three priorities as ‘mutually reinforcing’ (EC, 2018: 8).

In the same vein, the European Green Deal reaffirms the commitment to ‘sustainable and inclusive growth’ (EC, 2019: 2). The Green Deal is

‘a new growth strategy that aims to transform the EU into a fair and prosperous society, with a modern, resource-efficient and competitive economy where there are no net emissions of greenhouse gases in 2050 and where economic growth is decoupled from resource use’ (EC, 2019b: 2).

Its ‘ambition’ is to make Europe ‘the first climate-neutral continent by 2050’, while at the same time ‘creating new businesses, new jobs and triggering more investment’ (EC, 2020a: 1).

Despite the emphasis put on the innovative character of this policy approach, the Commission highlights the continuity with past efforts: ‘competitive sustainability has always been at the heart of Europe’s social market economy and should remain its guiding principle for the future’ (EC, 2019a: 3; see also EC, 2016: 2). The goal is to ‘ensure that Europe *remains* the home of the world’s most advanced welfare systems and is a vibrant hub of innovation and competitive entrepreneurship’ (EC, 2020a: 1, emphasis added). In this way, the Commission highlights at the same time the historical roots of the European Model, its relevance for the present (the high quality of life that it entails) and the will to project it into the future:

European integration and EU policies have helped to overcome post-war poverty and famine, and have created a space of liberty and democracy where European citizens could reach unprecedented levels of prosperity and well-being. (. . .) Our social market economy has generated prosperity and provided security thanks to strong welfare systems. (. . .) Furthermore, the EU has set some of the highest social and environmental standards, has put in place some of the most ambitious policies to protect human health, and has become the global champion in the fight against climate change. The EU Member States have achieved remarkable progress in many areas of the United Nations 2030 Agenda, and as a result, the EU is one of the best places in the world to live in, if not the best (EC, 2019c: 6)

Yet, this highly successful model is presented as being endangered: ‘there are many challenges that have become increasingly pressing, and threaten our well-being and economic prosperity’ (EC, 2019c: 10). Sometimes, the challenges are generated precisely by Europe’s success. For example, while it is ‘a great achievement’ that Europe has ‘the highest life expectancy in the world’, population ageing ‘brings its own challenges for our socioeconomic model’, especially ‘a deep impact on public finances, including on health systems’ (EC, 2019c: 12).

Thus, the current historical conjuncture is framed as a ‘moment of transformation’ and – in face of these multiple challenges – ‘Europe must act to avoid decline’ (EC, 2010: 5–6). There is, moreover, a sense of ‘urgency’, especially in tackling environmental problems (such as climate change), which require ‘immediate and decisive’ interventions (EC, 2018: 2): ‘urgent action’ is needed and the time to confront the sustainability challenge is ‘now’ (EC, 2001: 4). While we have the capacity to stop global warming and the loss of ecosystems and biodiversity, ‘we do not have the luxury of time’ (EC, 2019c: 7). But this sense of *urgency* also applies to tackling various social issues. For example, the need to ‘improve and adapt skills’ is framed as a pressing ‘imperative’: ‘Now, more than ever, the EU needs a paradigm-shift on skills. One that delivers a bold skills agenda for jobs’ (EC, 2020b: 1).

The good news is that since all these challenges are ‘strongly interlinked’, ‘addressing one may have positive implications for others’ (EC, 2019c: 10).

Crucially, the European Model should be reformed in order to face those challenges while *maintaining its core unchanged*: the goal is to ‘make sure that Europe’s Social Market Economy continues to create more and better jobs for all’ (EC, 2020a: 6). Hence, although the Commission recognizes the need for ‘transformational change’ (EC, 2014a: 4), the latter ultimately boils down to the modernization of the Model: the goal is to ‘upgrade Europe’s social market economy to fit the opportunities and challenges of today and tomorrow’ (EC, 2020a: 3). Europe ‘needs to modernise’ in order ‘to maintain a momentum that mutually reinforces economic growth, social welfare and environment protection’ (EC, 2005: 3). In the same vein, the vision – in itself potentially radical – of ‘living well within the limits of our planet’ then boils down to making a ‘smarter use of resources’, fostering green employment and using low-carbon technologies:

The EU has already embarked on this transition. Between 2000 and 2015, employment grew at a faster rate in the environmental sector than in the economy overall. Low-carbon technologies are becoming a major trade commodity, with the EU benefitting from significant positive trade balances. During the period 2012-2015, EU exports of clean energy technologies reached EUR 71 billion, exceeding imports by EUR 11 billion. The EU is already showing that it is possible to grow the economy, and reduce carbon emissions at the same time. (EC, 2019c: 14)

In this process of modernization, technological innovation plays a key role. There is a strong optimism that innovative environmentally friendly technologies, renewable resources and energy efficiency (EC, 2001: 2, 2010: 4) will allow decoupling of environmental degradation and resource consumption from economic growth. Moreover, improving ‘resource efficiency’ would not only ‘significantly help limit emissions’ but also ‘save money and boost economic growth’ (EC, 2010: 13). The political possibility of continuing the European economic and growth strategy and getting rid of ecological problems depends entirely on the technological realization of tremendous increases in energy and resource efficiency. This faith in technological solutions and win-win strategies reinforces the approach of economization that characterizes the European Social Model in its dealing with social inequality and social risks. Solutions for the social as well as the ecological question are not a political matter that requires open democratic discussions and structural changes in the social forms of living together. Rather, scientific evidence on various socioeconomic and environmental trends is enough to formulate policy responses. In this way, the future is foreclosed from political debate. The ‘challenges’ and dynamics that are transforming European societies are treated as exogenous, and the policy ‘solutions’ are self-evident exactly like the problems they have to address.

To be sure, the Commission is very keen to promote the participation of citizens, civil society organizations and relevant stakeholders. Yet, participation is mainly encouraged with a view to enhancing the acceptability and legitimacy of the ‘transition’: since the ‘destination’ of the transition is already defined ex-ante by the Commission, participation is not about defining the goals and final ends of public action but on finding the best and most efficient means to realize the already established objectives. As the Commission puts it: ‘Since it [the transition] will bring substantial change, active public participation and confidence in the transition is paramount if policies are to work and be accepted’ (EC, 2019b: 2). Similarly, addressing inequalities is crucial for the public to support the sustainability transition (EC, 2019c: 12). Indeed, inequality threatens social cohesion (EC, 2020a: 2) whereas ‘a socially fair transition is crucial to ensure a politically feasible transition’ (EC, 2018: 23). On this basis, a lot of emphasis is put on the need to make the transition ‘just’, ‘inclusive’, ‘fair’, supporting especially the ‘most vulnerable’ citizens, those populations ‘exposed to the harmful effects of climate change and environmental degradation’ and the ‘regions and sectors that are most affected by the transition because they depend on fossil fuels or carbon-intensive processes’ (EC, 2019b: 16; see also EC, 2018: 19, 2020a: 5). The Commission also connects the increasing sense of uncertainty and injustice (‘many Europeans do not feel well protected in a world that seems to them increasingly unfair’) with the ‘rising temptations of isolationism and nationalism’ (EC, 2019c: 12). Overall, it seems that the main concern is to secure ‘social cohesion’ and political stability rather than to seriously open the meaning of ‘transition’ to political debate. The goal is to make ‘socially acceptable’ the necessary ‘deep modernisation process’, but the latter is framed mainly as a technocratic matter that needs to be ‘managed well’ (EC, 2018: 20). In this way, larger questions concerning the kind of society we want to build in the face of social and ecological challenges remain marginalized. Only ‘managed’ forms of participation are encouraged – those that do not threaten the status quo and its power structures.

## Reconciling economic and social-ecological goals through economization

Perhaps the most important defining characteristic of the European Eco-Social Model is that, in line with the idea of sustainable development, the goals of economic growth, social inclusion and environmental protection ‘must go hand-in-hand and mutually reinforce one another’ (EC, 2005: 41). It is possible to distinguish two sides in the arguments of the Commission, which correspond to two ‘roads’ that connect the economic field with the social and ecological spheres. The first road goes from the economy to the ecological and social domains. In this context, the Commission emphasizes that a strong economy is essential for promoting both social and ecological goals: a strong economy will ‘generate the means to invest, for example in a cleaner environment, in better education and health care and in social protection’ (EC, 2005: 4). The second road goes the other way around, as ‘more sustainable use of natural resources and increased social justice are critical to our economic success’ (EC, 2005: 4). Taken together, these two roads constitute a virtuous, self-reinforcing circle: since investing in social and ecological goals delivers also economic returns, even more resources are made available for future investments. And, in fact, the ‘EU economy is expected to more than double by 2050 compared to 1990 even as it fully decarbonises’ (EC, 2018: 19).

Crucially, the fact that the social, ecological and economic dimensions are reconciled does not mean that they are equally important. Indeed, it seems that economic goals should take precedence over other goals. For example, among the ten priorities of the Juncker Commission, ‘jobs, growth and investment’ is the first priority, whereas ‘fundamental rights’ is only the seventh priority – coming after issues related to the single market, open trade and the monetary union – and ‘democratic change’ is the last priority (EC, 2019c: 47). Moreover, the central element that makes the self-reinforcing circle working is the *economization* of not only social but also ecological goals, that is, the extension of the economic logic to the social and ecological spheres. For example, the natural environment is reframed as ‘natural capital’ (e.g. EC, 2019b: 13) and valued in terms of the ‘ecosystem services’ (e.g. EC, 2019c: 14), that is the flow of benefits that nature delivers to people and the economy. Similarly, the promotion of environmental goals is seen as an opportunity for increasing economic and employment growth and enhancing competitiveness: applying ‘circular economy principles’ in all sectors will generate ‘a net economic benefit of EUR 1.8 trillion by 2030’ and ‘result in over 1 million new jobs across the EU by 2030’ (EC, 2019c: 16). Reducing ‘materials input through re-use and recycling’ will ‘improve competitiveness, create business opportunities and jobs, and require less energy’ (EC, 2018: 12). In this context, the shift ‘to a green and resource efficient economy is *above all* an opportunity to increase European global competitiveness’ (EC, 2014b: 13, emphasis added). The political strategy of subordinating social issues to a growth-centred economy is now being replicated in the integration of ecological issues. What has led to the model of a social investment state in social policy is now to guide an environmental investment policy in ecological issues: The investment in human capital (via education) and natural capital (via technology) forms the bracket of the approaches in the former European Social Model and the new European Eco-Social Model.

There are several declinations of this general process of extending the economic logic to non-economic domains. In the following, we analyze in more detail three forms of economization: emphasizing the economic costs of social-ecological problems and using market-based instruments for including these costs in market interactions, focusing eco-social policies on labour market empowerment and expanding profit-making opportunities for the private sector.

### Framing socio-ecological problems as economic costs and using market-based instruments

A first form of economization involves the reframing of social-environmental problems in terms of economic costs and the use of the market mechanism for reducing them. Concerning the economic costs of unsustainability, the Commission highlights how climate change causes costly natural disasters (such as river floods), aridity and the decline of (outdoor) labour productivity – with all costs precisely quantified in monetary terms (EC, 2018: 2). Similarly, environmental problems such as air pollution imply an ‘health damage by around € 200 billion per annum’ (EC, 2018: 16) and the ‘annual loss of ecosystem services is estimated equivalent to €50 billion, while by 2050 the cumulated welfare losses were estimated equivalent to 7% of GDP’ (EC, 2009: 7). The same costs-centred logic applies to ‘social’ problems, which are reframed as obstacles to economic growth: ‘Inequalities of opportunity can impede parts of the population from social and labour market inclusion, hampering growth prospects’ (EC, 2019c: 12).

Given these high costs, promoting sustainability should actually be economically rational. The problem is that the market mechanism is not always able to reflect these costs. Yet, the solution for this market failure consists in integrating these negative externalities in the market: the solution is more market rather than less. Thus, the Commission prioritizes a market-based approach that further expands the market sphere (and its logic) into new domains. One crucial market-based instrument is the ‘Emissions Trading System’, which ensure ‘effective carbon pricing throughout the economy’, encouraging ‘changes in consumer and business behaviour’ and facilitating ‘an increase in sustainable public and private investment’ (EC, 2019b: 5).

The basic idea behind market-based approaches is that ‘prices have a powerful influence on the behaviour of individuals and businesses’ and that market reforms ‘to get prices right’ (e.g. removing subsidies that encourage wasteful use of natural resources and putting a price on pollution) can ‘create new business opportunities to develop services and products that ease pressure on the environment and fulfil social and economic needs’: changing prices in this way ‘provides a permanent incentive for the development and use of safer, less polluting technologies and equipment, and will often be all that is needed to tip the balance in their favour’ (EC, 2001: 7).

Indeed, according to the Commission, ‘the most powerful method to promote change is to ensure that markets send the right signals’ so that both producers and consumers face the full costs of their decisions: integrating the ‘cost imposed on others in society by “polluters” into the price of the product’ (e.g. through green taxes), ‘producers have an incentive to produce and consumers an incentive to consume more environmentally-friendly goods’ – and sometimes it is enough to provide information (e.g. labelling) to help consumers choose these goods (EC, 2005: 16).

### Focusing eco-social policies on labour market empowerment

Another form of economization consists in focusing eco-social policies on labour market empowerment, i.e. labour market participation and productivity-enhancement. For example, education is framed as an ‘investment in human capital’ (e.g. EC, 2020b: 21). In this context: ‘Skills and lifelong learning are crucial for long-term and sustainable growth, productivity and innovation and therefore a key factor for the competitiveness of businesses’ – indeed ‘It is only with the right skills that Europe can strengthen its position in global competition’ (EC, 2020b: 2). Thus, in largely instrumental way, education is mostly reduced to a matter of enhancing those ‘skills’ relevant for ‘labour market participation’ (EC, 2010: 4). As the Commission puts it (EC, 2020a: 4): in order to find out ‘what skills we need’, governments ‘have to work with those who know best: employers, workers, teachers and trainers’ – and there are few doubts that these actors appear in the list in order of importance. In fact, the Commission continues, in a ‘volatile and changing job market, it is crucial that all people possess broad key competences that lay a good foundation for their capacity to adapt’ (EC, 2020a: 4). In other words, education should contribute to the *adaptability* of the population to the needs of companies: the goal is to ‘foster employability’ and ‘meet the changing needs of businesses’ (EC, 2020a: 4).

Crucially, this investment in people’s human capital

‘pays off: 1 euro invested in up- and reskilling returns at least 2 euros in revenues or savings (EC, 2020b: 23). The fact that investments in skills have an economic return suggests that these investments can (and should) become attractive also for the private sector. The Commission indeed proposes to use ‘social impact bonds’

in order ‘to boost investment in skills’ (EC, 2020b: 24). With social impact bonds, private investors can make a profit on investments in the provision of social services (such as training provision). This points directly to the last and most radical form of economization, which implies that social-ecological issues are transformed into profit-making opportunities for the private sector.

### Generating profit-making opportunities for the private sector

The Commission emphasizes the importance of the private sector for the transition. In fact, the private sector ‘remains the key driver of inclusive and sustainable growth’ (EC, 2014a: 14), especially through ‘social entrepreneurship’ (EC, 2019c: 27) and ‘eco-entrepreneurship’ (EC, 2014a: 9). Promoting ‘inclusive and sustainable growth’ – that is moving towards an ‘inclusive green economy’ in which ‘the benefits are widely shared’ – requires, among others, to build ‘market-friendly, open economies’, to ‘improve productive capacities’ and to ‘promote private sector development, investment and wealth creation’ (EC, 2013: 9). Indeed:

the private sector, ranging from small stakeholders to major multinationals, is an important engine for innovation, sustainable growth, job creation, trade and poverty reduction. It also plays a critical role in investing in resource efficiency and infrastructure, such as sustainable transport systems, energy networks and digital infrastructures that are vital for a country’s economic growth. Implementing the post-2015 agenda therefore requires a business environment that is conducive to private sector initiatives (EC, 2015: 12).

In this context, the policy approach privileged by the Commission is that of ‘encouraging a greater sense of corporate social responsibility and in establishing a framework to ensure that businesses integrate environmental and social considerations in their activities’ (EC, 2001: 8). In line with the market-based approach described above, public policies should ‘enhance market reward for corporate social and environmental responsibility’, ‘disseminate good practice’, ‘improve self and co-regulation processes’ and ‘improve company disclosure of social and environmental information’ (EC, 2015: 13).

However, while public policies should establish an enabling framework for corporate social responsibility, the Commission highlights that some of ‘the most far sighted businesses have realized that sustainable development offers new opportunities and have begun to adapt their investments accordingly’ (EC, 2001: 8): more and more companies ‘see the SDGs as an integral part of their competitiveness and growth strategy’ since ‘they have understood that responsible business can lead to more sustainable profits and growth, new market opportunities, and long-term value for shareholders’ (EC, 2019c: 26). Investing in sustainability ‘pays’, as it ‘spurs investment in new technologies, processes and products’, therefore, it ‘makes good business sense’ to ‘plan ahead’ and ‘invest for the future’ (EC, 2005: 17). The Commission even reports certain estimates according to which ‘achieving the SDGs in the areas of food, agriculture, energy, materials, cities, and health and well-being could open more than EUR 10 trillion of market opportunities’ (EC, 2019c: 14).

Moreover, not only companies but also the ‘financial sector has a key role to play in supporting the transition towards net-zero emissions as it can reorient capital flows and investments towards the necessary solutions while improving efficiency of production processes and reducing the cost of financing’ (EC, 2018: 18). Thus, it is essential to reorient private capital to more sustainable investments, including through ‘sustainable finance’ and ‘green bonds’ (EC, 2019c: 24).

**Figure 1.**
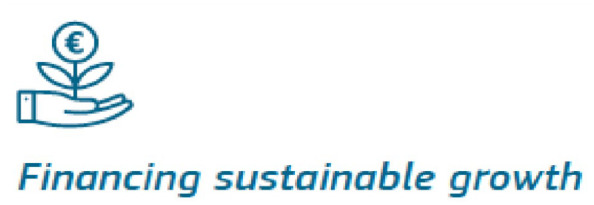
Financing sustainable growth. Source: EC (2019c: 50).

This economization form is well symbolized by the image used in one document (EC, 2019c: 50) for illustrating the financing of sustainable growth, where, instead of flowers, eurocoins grow out of plants (Figure 1). This image suggests that investments in green growth deliver financial returns and implies a rather instrumental and economistic view of nature as a means for increasing profits, thereby inverting means and ends in a way typical of ‘economization’ processes. Indeed, an image representing plants and flowers growing out of money would have been the symbol of an economy, and a financial sector subordinated to nature and in the service of environmental sustainability – but here it is clearly the other way around: the ‘natural capital’ is an investment object that should generate profits for the financial sector. The ecological transition becomes a ‘unique business opportunity’ (EC, 2018: 9): ‘investing into a sustainable society’ (EC, 2018: 15) transforms social-ecological goals from ends into means, whereas money becomes the end rather than the means.

## Beyond economization?

The enhancement of the European Social Model to a European Eco-Social Model as it emerges from the documents of the Commission relies on the reconciliation of economic, social and ecological goals through economization. Yet, there are some elements in the documents that, although less present, seem to indicate a different direction. For example, at one point, the Commission argues that ‘Economic growth is not an end in itself’ and that ‘An economy must work for the people and the planet’ (EC, 2019a: 1). If this perspective were fully embraced, it would reverse the economization processes that invert the relationship between means and ends. Such an approach would establish clear priorities, with social-environmental goals taking precedence over other concerns and with an economy subordinated to and in service of those goals.

Similarly, the framework of economizing social and ecological issues could be challenged if it is given more room to the ‘rights based-approach’ endorsed by the Commission, which aims to reducing inequalities and giving people – and especially ‘marginalized and vulnerable groups’ – a ‘say in policy choices and decision-making that affect them’ (EC, 2014a: 10). The Commission also recognizes the problems associated with a technocratic policymaking informed mainly by ‘science and scientific advice’ and biased towards the economic elites:

There are concerns that the policy responses have been driven more by narrow sectional interests than the wider interests of society. (. . .) Many believe that policy has become too technocratic and remote, and is too much under the influence of vested interests. To tackle this rising disaffection with the political process, policy making must become more open. An open policy process also allows any necessary trade-offs between competing interests to be clearly identified, and decisions taken in a transparent way. Earlier and more systematic dialogue (. . .) may lengthen the time taken to prepare a policy proposal, but should improve the quality of regulation and accelerate its implementation. The views of those from outside the Union should also be sought (EC, 2001: 8).

However, these elements pointing to an alternative to the status quo and to transformative change remain underdeveloped in the documents and even in those cases where the problematization appears radical, the solutions become subordinated again to the logic of economization. For example, shortly after having recognized that ‘our culture of consumption has resulted in excessive resource extraction and growing pressures on natural capital and climate’, the Commission argues that ‘We need to make sure that we can continue to grow our economy in a sustainable way and improve the living standards people demand’ (EC, 2019c: 15).

To be sure, the presence of contradictions in policy documents is not a surprise and fully understandable given the fact that these documents are the result of compromises and negotiations among actors with very different worldviews and interests. The problem then is not so much the incoherence of the argumentation but rather that some argumentative lines tend to systematically prevail over others. The different weights given to the specific arguments actually reveal the persistence influence of powerful actors – of their worldviews and interests. From this perspective, the commitment by the Commission (EC, 2001: 5) to oppose powerful interests (‘narrow sectional interests must not be allowed to prevail over the well-being of society as a whole’) appears largely rhetorical. On this basis, it seems that the presence in the texts of discourses and narratives that are potentially at odds with the overall economization-centred approach proposed by the Commission is a way of neutralizing opposition and de-politicizing conflicts.

## The global role of the European Eco-Social Model

The documents analyzed allowed us not only to identify the main characteristics of the European Eco-Social Model but also to detect the global role that the EC envisions for this Model. Indeed, the Commission insists a lot on the special role of the EU in global policy. The reason for this is that Europe ‘is a unique place where prosperity, fairness and a sustainable future are equally important goals’ (EC, 2020a: 1). The EU was also one of the leading forces behind the United Nations 2030 Agenda (EC, 2019c: 6) and this Agenda ‘is fully consistent with Europe’s vision’ (EC, 2016: 3). Thus, EU Member States ‘are among the happiest countries in the world and among the SDG top performers’ (EC, 2019c: 6-7). On this basis, the EU is framed as a ‘global leader’ (EC, 2019c: 20) and a ‘global player’ that ‘takes its international responsibilities seriously’ (EC, 2010: 22).

This European leadership should be developed ‘along twin internal and external tracks’ (EC, 2005: 40), that is promoting sustainable development both internally (in the EU) and externally, providing support to other countries and at the global level. It seems that these two ‘tracks’ are interlinked and inseparable. On the one hand, in ‘an ever more globalized world’, the very possibility of a European Model (the internal track) demands global leadership (the external track): ‘clear political leadership is necessary to promote a dynamic European Model for today and in the future’ (EC, 2005: 40). On the other hand, the European Model can become an example for the rest of the world. Thus, the goal of making the EU the first to achieve net-zero greenhouse gas emissions by 2050 (internal track) in itself already implies to ‘lead the way worldwide’ (external track): ‘demonstrating that net-zero emissions can go hand in hand with prosperity’, Europe will have ‘other economies follow its successful example’ (EC, 2018: 22–23).

The internal and external ‘tracks’ appear interlinked also because, as ‘the world’s largest single market’, the ‘EU’s high environmental standards on products have effects that go far beyond the borders of the EU’ – and the same applies to the EU’s trade policy, which contributes to sustainable development both in the EU and in third countries (EC, 2018: 21–22). Indeed: ‘Open and rules-based trade is one of the best tools to increase our prosperity and that of our partners, raise our standards of living and the sustainability of our planet and our democracies’ (EC, 2019c: 27).

This optimism concerning the role of international trade in promoting sustainable development (see also: EC, 2015: 9–11) is complemented by the commitment to a ‘rules-based multilateral world order’, which is seen as ‘the best antidote to the law of the jungle in an anarchic world rife with nuclear weapons, extremism, and limited resources’ and for opposing the ‘growing dangerous nationalist strain of ‘my country first’’ that can lead to conflict and to the withdrawal from ‘global commitments to human welfare, security, environmental protection and climate action’ (EC, 2019c: 8). Therefore, ‘We do not need more walls, but global rules respected by all’: ‘The rules-based system is the best guarantor for the sustainability of our economy and society’ (EC, 2019c: 31).

On this basis, there is the recognition that ‘the EU’s long-term strategy cannot be pursued in isolation’: the ‘EU efforts in leading a successful low-carbon transition at global level and fighting climate change ultimately depend on international cooperation’ (EC, 2018: 21). Thus, efforts should focus ‘on convincing and supporting others to take on their share of promoting more sustainable development’ – with the EU ‘setting a credible example, and following-up with diplomacy, trade policy, development support and other external policies’ (EC, 2019c: 20).

But beyond the importance of being an example for others, the Commission also strongly emphasizes the rewards of this role of global leader in terms of ‘the benefits of first mover advantage’ (EC, 2018: 5). Taking the lead in the transition to a sustainable economy, the EU ‘can set the standards for the rest of the world’, being ‘the first to reap the benefits of the transition’ and having ‘the strongest competitive advantage in the global marketplace of tomorrow’ (EC, 2019c: 14). In particular, the EU can ‘position itself as a world leader in eco-efficient technologies’:

There is a growing realisation – not least among business – of the scale of opportunity to be seized in investing in eco-innovation. The market for sustainable products and processes will have to grow to meet the demands of a fast growing global ‘middle’ class, for consumer goods and services alongside environmental quality. A coordinated approach, anticipating the need to shift to more sustainable production and consumption process, will provide Europe with a competitive edge. (EC, 2005: 9)

The transition to clean energy and to an economic system in which energy supply largely comes from renewable energy sources would also imply the reduction of Europe’s energy import dependence (especially oil and gas imports) and this in turn would not only improve the EU’s ‘geopolitical position’ but also foster ‘domestic jobs’ and generate savings (‘€ 2-3 trillion over the period 2031–2050’), thereby ‘freeing resources for further potential investments into the modernisation of the EU economy’ (EC, 2018: 8–9). Similarly, becoming ‘greenhouse gas emissions free’ is framed as an ‘investment’ essential for successfully benefitting from ‘the next industrial revolution’: the ‘modern, competitive and prosperous EU industry, by staying at the forefront of the transition, would be able to strengthen its presence in a global economy that will inevitably become increasingly carbon constrained’ (EC, 2018: 12).

Another aspect that emerges from this global leadership role is a subtle form of ‘arrogance’, whereby the Commission appears quite self-confident to know what is best for the rest of the world – an attitude that could be potentially labelled as *neo-colonialist*:

The EU and the United Nations are natural partners in the efforts to shape a safer and better world for all. (. . .) Ultimately, to be most successful in the green and inclusive economic transition, we have to get our global partners on board too and make the case that a global sustainable development model based on our core values and principles is the best way to achieve shared prosperity and a sustainable world (EC, 2019c: 31)

Thus, there is the assumption that the European Model *can* and *should* be ‘exported’ to the rest of the world. However, while it is far from clear that the European way of living is extendable to the whole world without generating an ecological collapse, it is also not obvious that all peoples or countries are interested in pursuing this way or in accepting those (apparently superior) European values. This arrogance vis-à-vis the rest of the world – and of course especially towards the Global South – is complemented by a very limited sense of global justice. For example, there is no mention of the fact that Europe is more responsible of environmental degradation than poor countries and no obligations seem to emerge from the colonial past (in the sense of restorative justice). Instead, the Commission highlights the generosity of the European countries as donors for developmental aid (EC, 2019c: 31).

Apart from the fact that the position of ‘donor’ is again one of power and superiority – which is very different from adopting the perspective of restorative justice – donations involving a percentage of GDP below one are a good indicator of the relevance of any ideas of global justice within the European project.

## Conclusion

The empirical analysis of the most relevant documents adopted by the EC since 2000 for framing together eco-social and economic issues reveals that the main characteristic of the European Eco-Social Model is that of attempting to reconcile socio-ecological and economic goals through economization. Thus, what was true for the social dimension of the European project applies also to its ecological dimension: *environmental goals, like social goals, are framed in a way that subordinates them to the economic rationality* of growth and competitiveness.

Economization implies that technological innovation is seen as the central factor allowing to decouple economic growth from resource use and environmental degradation. In this context, there are pre-defined challenges and opportunities and the ‘solutions’ – like the ‘problems’ – are presented as being mostly self-evident and uncontroversial. Economization takes three main forms. First, the economic costs of ecological problems are emphasized. Market-based instruments are then proposed for making sure that the price mechanism reflects these costs, providing incentives to both producers and consumers to opt for environmentally friendly goods. Thus, the solution to market failures such as negative externalities is not searched in the limitation but – paradoxically – in the promotion of the market. Second, shifting to a green economy should become a motor of job creation – which requires to conceive social policies in terms of their capacity to enable people to work (e.g. education becomes a human capital investment). Third, ecological goals are reframed as economic goals that should generate economic returns – including profits for the financial sectors. Parallel to investing in human capital, investments should also be made in natural capital. Environmental and social problems are transformed into investment objects that promote economic growth. Finally, the analysis also reveals that the Commission believes that this modernized European Eco-Social paradigm should become a model to follow for the rest of the world. Thus, while the ‘solutions’ advanced by the Commission are not specifically European – they are largely shared by international organizations and national countries worldwide – the Commission portrays this strategy as a specifically European one and as a successful model to be exported.

On the basis of this study, the political project developed by the Commission can be criticized for promoting those economization processes. Indeed, it is far from clear that this ‘model’ can fulfil its promises. While there are important doubts concerning the very possibility of green growth (Hickel and Kallis, 2020), the project of extending the European Model to the whole world is based on an internal contradiction. The EU’s selected solution to ecological problems should become established worldwide; at the same time, the EU sees itself as a global leader. But if the other countries of the world follow this model with its demands for prosperity and growth, the ecological problems will increase further, so even greater efforts would be needed to cope with them technologically. In the end, it is hoped to be one step ahead of all others in order to maintain European leadership – and set more and more incentives for further ecological degradation. The model promotes a vicious cycle of increasing economization and is thus internally naïve. Moreover, it fails to address questions of global justice – whichever version of a theory of global justice one takes as a starting point (Brooks, 2020).

From this perspective, probably the only possibility to move to a paradigm centred on environmental sustainability is to rethink social policy beyond productivism in the direction of *post-growth welfare politics* (e.g. Büchs, 2021; Dukelow and Murphy, 2022; Fitzpatrick, 2004; Hirvilammi, 2020; Koch, 2022). While some – underdeveloped and marginalized – elements of this alternative path are present in the documents by the Commission (see also Ossewaarde and Ossewaarde-Lowtoo, 2020), they are subordinated to the dominant strategy centred on economization. As Samper et al. (2021: 14) argue with respect to the European Green Deal, the Commission attempts to incorporate some of the ‘counterhegemonic narratives’ with a view to eliminate the antagonism and contestation inherent in eco-social politics, thereby foreclosing the democratic articulation of opposition. Thus, our findings are in line with the recent work suggesting that the policies promoted at the European level so far are insufficient for addressing the social and ecological demands of our time (Crespy and Munta, 2023; Eckert and Kovalevska, 2021; Zimmermann and Gengnagel, 2023). More generally, we agree with Schulze Waltrup et al. (2023) on the need to understand eco-social policies in the context of a broader global political economy dominated by capitalist imperatives – and on the need to overcome the latter. Rather than promoting social and ecological goals through their economization, what is needed is the subordination of the economy to the satisfaction of democratically-defined social and ecological needs.
